# Intermittent Hypoxia Exposure Can Prevent Reductions in Hemoglobin Concentration After Intense Exercise Training in Rats

**DOI:** 10.3389/fphys.2021.627708

**Published:** 2021-02-19

**Authors:** Xiquan Weng, Hao Chen, Qun Yu, Guoqing Xu, Yan Meng, Xu Yan, Glenn McConell, Wentao Lin

**Affiliations:** ^1^Department of Exercise Biochemistry, College of Exercise and Health, Guangzhou Sport University, Guangzhou, China; ^2^College of Sport, Yancheng Teachers University, Yancheng, China; ^3^Institute for Health and Sport, Victoria University, Melbourne, VIC, Australia; ^4^Australia Institute for Musculoskeletal Sciences, Melbourne, VIC, Australia

**Keywords:** hypoxia, IHE, erythropoietin, EPO, hemoglobin

## Abstract

Intense exercise training can induce low concentrations of hemoglobin, which may be followed by maladaptation. Therefore, it is important for athletes to prevent low concentrations of hemoglobin during intense exercise training. In this study, we explored whether different protocols of intermittent hypoxic exposure (IHE, normobaric hypoxia, 14.5% O_2_) could prevent the exercise training-induced reduction in hemoglobin concentration in rats. Six-week-old male Sprague-Dawley rats were subjected to progressive intense treadmill exercise training over three weeks followed by three weeks of training with IHE after exercise. IHE lasted either 1 h, 2 h, or 1 h + 1 h (separated by a 3-h interval) after the exercise sessions. Hematological parameters, including hemoglobin concentration [(Hb)], red blood cells (RBCs), and hematocrit (Hct), and both renal and serum erythropoietin (EPO) were examined. We found that intense exercise training significantly reduced [Hb], RBCs, Hct, food intake and body weight (*P* < 0.01). Analysis of reticulocyte hemoglobin content (CHr) and reticulocyte counts in the serum of the rats suggested that this reduction was not due to iron deficiency or other cofounding factors. The addition of IHE after the intense exercise training sessions significantly alleviated the reduction in [Hb], RBCs, and Hct (*P* < 0.05) without an obvious impact on either food intake or body weight (*P* > 0.05). Increase in reticulocyte count in the rats from the IHE groups (*P* < 0.05 or *P* < 0.01) suggests that IHE promotes erythropoiesis to increase the hemoglobin concentration. Furthermore, the addition of IHE after the intense exercise training sessions also significantly increased the concentration of renal EPO (*P* < 0.05), although the increase of the serum EPO level was statistically insignificant (*P* > 0.05). The different IHE protocols were similarly effective at increasing renal EPO and preventing the training-induced decreases in [Hb], RBCs, and Hct. Collectively, this study suggests that IHE may be used as a new strategy to prevent intense exercise training-induced reductions in [Hb], and deserves future exploration in athletes.

## Introduction

Sport training is a process of carefully applied stress-adaptation (Lorenz and Morrison, 2015; Haugen et al., 2019). Providing stress at the optimal level that an athlete can endure will lead to improvements in physical function and performance (Lorenz and Morrison, 2015; Haugen et al., 2019). In contrast, stress beyond the optimal level will lead to maladaptation and may eventually lead to fatigue or even overtraining (Kellmann, 2010). Therefore, seeking appropriate monitoring, nutritional, and rehabilitation strategies during periods of intense exercise training is critical to help athletes to avoid excessive stress, and to improve physical function and performance.

Blood hemoglobin (Hb) is a routinely used marker for monitoring intense exercise training and physical function (Gleeson, 2002; Halson et al., 2003). Based on previous studies, it has been accepted that a 10% decrease in hemoglobin concentration [(Hb)] can be a practical indicator to predict maladaptation caused by intense exercise training, although this state can result from plasma dilution, which frequently occurs early in prolonged training process (Zhao, 2003; Sheng, 2006). During intense exercise training, the [Hb] of the athletes of low concentration of hemoglobin (at least 10% drop of [Hb)] will keep dropping if they continue to participate the training, which will impair their ability to endure the intense exercise training (Zhao, 2003; Sheng, 2006). Therefore, it is particularly important to avoid low concentrations of hemoglobin by promoting the synthesis of Hb through appropriate strategies.

Currently, altitude training (hypoxic training) is widely used to improve the aerobic capacity of athletes (Millet et al., 2010). Hypoxia can stimulate erythropoietin (EPO) secretion under the regulation of hypoxia inducible factors (HIFs) (Haase, 2013; Ploszczyca et al., 2018; Viscor et al., 2018). HIFs consist of a heterodimer of an oxygen-sensitive α-subunit (HIF1α) and a constitutively expressed β-subunit (HIF1β) (Haase, 2013). Under normoxia, HIFα subunits are rapidly hydroxylated and subjected to either proteosome-mediated degradation or deprivation of transcriptional activity (Haase, 2013). Conversely, during hypoxia, the HIFα subunit is not hydroxylated, and dimerized with HIF1β to activate the transcription of target genes (Haase, 2013). Numerous studies demonstrated that the secretion of EPO induced by altitude training can increase Hb content, oxygen binding, and oxygen transport in athletes (Rodriguez et al., 2000; Ploszczyca et al., 2018; Viscor et al., 2018). For example, intermittent hypoxia exposure (hypobaric hypoxia at a simulated altitude of 4000–5500 m) for 90 min, three times a week for 3 weeks, significantly increased reticulocytes (180%), RBCs (7%), and [Hb] (13%) in athletes (Rodriguez et al., 2000). However, it is noteworthy that the erythropoiesis-promoted effect of altitude training is still under debate since certain studies showed negative results (Julian et al., 2004; Fu et al., 2007). Different outcomes in these studies may result from different experimental design, especially regarding to the dosage and the nature of the hypoxic stimulus.

So far, to our knowledge, hypoxia has not been specifically applied in promoting adaptations to intense exercise training of athletes. In this study, we explored the possibility of such an application in Sprague-Dawley (SD) rats since low concentration of hemoglobin also occurs in rats after intense exercise training (Zhu et al., 2010; Liu et al., 2011) and it seems more challenging to realize a large sample size and genetic homogeneity in athletes. We hypothesized that through intermittent hypoxic exposure (IHE) of a reasonable simulated altitude and a sufficient dosage, SD rats would not display low concentrations of hemoglobin after intense exercise training since hypoxia can stimulate EPO secretion which can subsequently facilitate erythropoiesis to increase Hb content based on human and rodent studies (Rodriguez et al., 2000; Cui et al., 2020). We investigated the effects of hypoxic exposure on the levels of [Hb], RBCs, hematocrit (Hct), and EPO (kidney and serum) in rats undergoing intense exercise training.

## Materials and Methods

### Animals and Diets

Six-week-old male Sprague-Dawley (SD) rats (*n* = 50, mean initial body weights = 158 ± 12g) were purchased from Guangdong Experiment Animal Research Institute (GEARI, Guangzhou, China). The rats were caged (5/cage) in a clean facility with a fixed light-dark cycle (12h/12h), a humidity of 40–60% and a temperature of 24°C, and had free access to fresh water and food purchased from GEARI. The iron content of the rat diet used in this study was 172.6 mg/kg, which should be enough to meet the iron demand of rats based on previous studies (Erikson et al., 2000; Staniek et al., 2020). All animal protocols were approved by the Guangzhou Sport University Animal Ethics Committee.

### Study Design

Fifty rats were assigned into 5 groups (10 per group): normoxic sedentary control (SC), normoxic exercise control (EC), 1-h hypoxia exposure after exercise (E + 1h), 2-h hypoxia exposure after exercise (E + 2h), and two separate 1-h hypoxia exposures after exercise [a 3-h interval between two hypoxic exposure, E + (1 + 1)h]. The design of different strategies of IHE aimed to explore the reasonable IHE periods (dosage) for preventing the exercising rats from displaying low concentrations of hemoglobin, while the addition of the E + (1 + 1)h group aimed to increase the number of intermittent hypoxia stimulations (from 1/day to 2/day) that might lead to a different effect from the (E + 2h) group due to the presence of an interval between two hypoxia stimulations. The rats in the EC, E + 1h, E + 2h, and E + (1 + 1)h groups undertook treadmill training sessions 6 days per week for 6 weeks. From the 4th to the 6th week, after the completion of exercise, the rats in Group E + 1h, E + 2h, and E + (1 + 1)h were exposed to normobaric hypoxia (14.5% O_2_) in a hypoxic chamber (Hypoxic Tent System, Hypoxico Inc., NY, United States) for their respective durations. The exercise session duration lasted for 10 min on the first day, and increased by 10 min per day thereafter until 1 h was reached and maintained for the remaining training days. In the first week, the exercise velocity commenced at 15m/min, and increased each week by 5m/min, to 40m/min in the sixth week.

### Sample Collection and Measurement of Hematological Parameters

Rats were anesthetized by using 50 mg/kg of sodium pentobarbital within 24 h after the end of the experiments. Blood samples were collected from the abdominal aorta into EDTA-containing or EDTA-lacking tubes. Immediately after collection, whole blood collected into EDTA-containing tubes was analyzed to determine [Hb], RBCs count, Hct, reticulocyte hemoglobin content, and reticulocyte count using an automated cell counter (ADVIA120, Bayer AG, Germany). The blood samples collected using EDTA-lacking tubes were centrifuged at 3,000 rpm/min for 20 min at room temperature to collect the serum for EPO measurements (see below). Kidneys were collected and homogenized to measure EPO (see below) as previously described (Garrido et al., 2015; Landau et al., 2018). The rats were then killed by an overdose of the anesthetic.

### EPO Measurement

Renal and serum EPO were measured using enzyme-linked immunosorbent assays (ELISA) according to the manufacturer’s guidelines (E02E0002, Bluegene Biotech CO., LTD, Shanghai, China). This assay applies the technique of quantitative sandwich enzyme immunoassay. A monoclonal antibody specific to rat EPO was pre-coated onto a microplate. Briefly, 100 μL of conjugate was added to each well in the plate, then 50 μL of standard, control, or sample was added to the plate and incubated for 1 h at 37°C. Each well was aspirated and rinsed with wash buffer for a total of five washes. Substrate (100 μL) was added to each well and incubated for 10–15 min at 37°C in dark. Stop solution (100 μL) was added to each well and the plate was read within 30 min. Plates were read at 450 nm on a VARIOSKAN FLASH (Thermo Fisher Scientific, MA, United States). Each sample was measured in duplicate.

### Statistics

The hematological and EPO data were analyzed using a one-way analysis of variance (ANOVA) followed by Bonferroni’s *post hoc* test. All statistical calculations were performed using IBM SPSS Statistics for Windows (version 19). *P*-values less than 0.05 were considered statistically significant.

## Results

**FIGURE 1:**
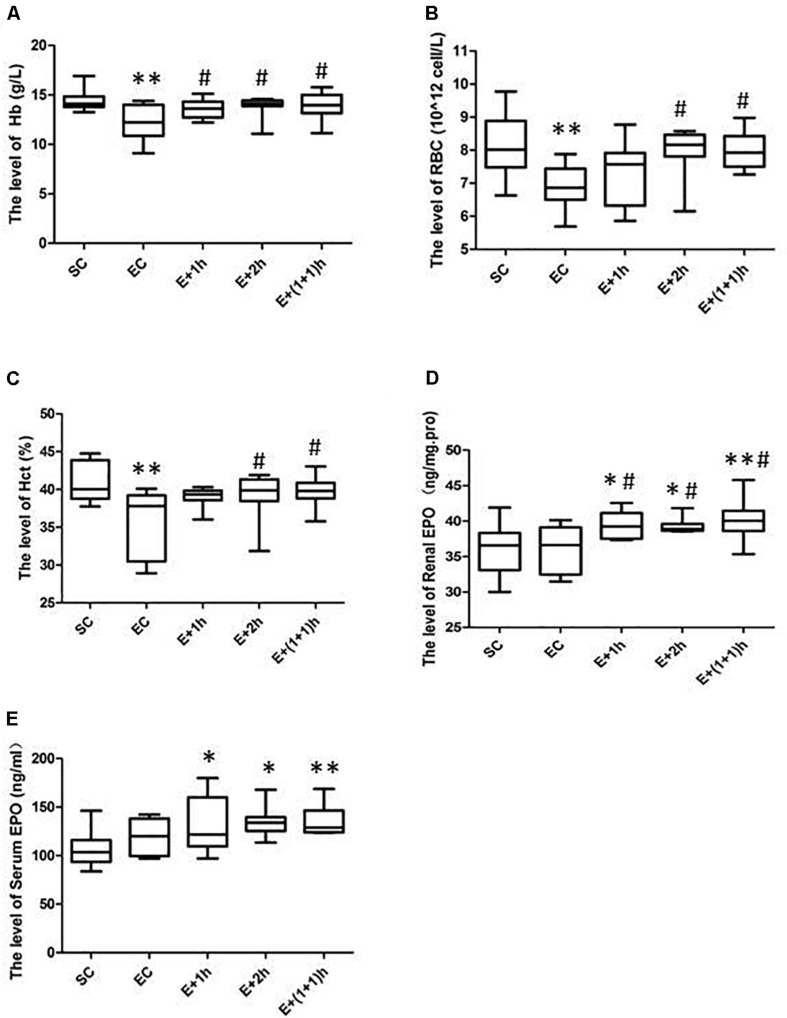
The hematological parameters and EPO levels for various groups. **(A)** Hemoglobin (Hb). **(B)** Red Blood Cells (RBCs). **(C)** Hematocrit (Hct) levels. **(D)** Renal erythropoietin (EPO) levels. **(E)** Serum EPO levels. The groups were normoxia sedentary controls (SC), normoxia exercise control (EC), exercise and 1-h hypoxia exposure (E + 1h), exercise and 2-h hypoxia exposure (E + 2h), and exercise and two one-hour hypoxia exposures [E + (1 + 1)h]. ^∗^Significantly different from SC, *P* < 0.05. ^∗∗^Significantly different from SC, *P* < 0.01. #Significantly different from EC, *P* < 0.05.

As shown in Figures 1A–C, the [Hb], RBC count, and Hct of the EC group all significantly decreased (*P* < 0.01) compared with those of the SC group for whom the [Hb] dropped ∼15%, indicating that the EC group displayed low concentrations of hemoglobin as expected. In addition, from the second week, the average daily food intake of rats in the EC group was significantly lower than that in the SC group (*P* < 0.01) (Table 1); from the 3nd week, the body weight of rats in the EC group was significantly lower than that in the SC group (*P* < 0.05, Table 2). These data suggested that the food intake and body weight of the rats in the EC group could have been affected by low concentrations of hemoglobin. The low concentrations of hemoglobin in the EC group may be caused by iron deficiency. To explore this possibility, we examined reticulocyte hemoglobin content (CHr or Ret-He), an early marker of iron restricted erythropoiesis (Mast et al., 2002; Roy et al., 2007; Torsvik et al., 2013). We found that the CHr of the EC group was significantly higher than that of the SC group (*P* < 0.01, Table 3). This result suggested that the low hemoglobin concentration in the EC group was not due to iron deficiency. To exclude the possibility that low concentrations of hemoglobin in the EC group was caused by cofounding factors such as water intake or manipulation stress, the reticulocyte counts of the rats which can reflects the erythropoietic activity of the bone marrow were examined (Cline and Berlin, 1963; Riley et al., 2001). Reticulocyte count can be reported as absolute reticulocyte count (Retic#) or as a reticulocyte percentage (Retic%) (Riley et al., 2001). After the IHE for three weeks, both the absolute reticulocyte count and reticulocyte percentage of the rats in the EC group were significantly higher than those in the SC group (*P* < 0.01 both, Table 4), which revealed that the low concentrations of hemoglobin in the EC group has promoted erythropoiesis. Therefore, it is less likely that the low concentrations of hemoglobin in the EC group were caused by temporary cofounding factors.

**TABLE 1:** The food intake (g) of rats before and during the experiments.

	SC	EC	E + 1h	E + 2h	E + (1 + 1)h
1st week	20.5 ± 2.4	20.5 ± 2.3	19.9 ± 2.0	20.9 ± 2.1	19.7 ± 2.3
2nd week	22.6 ± 1.1	19.0 ± 1.8**	19.5 ± 1.5**	20.3 ± 1.4**	20.2 ± 1.4**
3rd week	22.2 ± 2.0	17.9 ± 1.3**	18.5 ± 1.1**	18.6 ± 1.0**	17.8 ± 1.5**
4th week	23.4 ± 2.1	20.1 ± 2.3**	19.8 ± 2.5**	20.3 ± 2.4**	19.0 ± 2.7**
5th week	25.2 ± 2.8	19.0 ± 4.0**	18.9 ± 4.8**	18.4 ± 4.3**	20.1 ± 3.9**
6th week	26.2 ± 4.0	22.0 ± 5.3**	21.5 ± 5.8**	21.2 ± 6.4**	19.9 ± 7.1**

**Significantly different from SC, *P* < 0.01.

**TABLE 2:** The body weight (g) of rats before and during the experiments.

	SC	EC	E + 1h	E + 2h	E + (1 + 1)h
Before intervention	188.22 ± 9.65	187.05 ± 10.12	189.38 ± 11.36	185.59 ± 9.98	189.01 ± 10.88
1st week	210.78 ± 12.68	205.40 ± 19.23	205.66 ± 15.98	207.06 ± 17.97	209.65 ± 16.11
2nd week	237.14 ± 26.70	221.10 ± 30.58	219.26 ± 21.55	218.87 ± 26.31	222.18 ± 23.89
3rd week	256.63 ± 31.20	217.35 ± 26.90*	215.36 ± 25.57*	217.01 ± 25.32*	220.10 ± 27.32*
4th week	283.00 ± 32.42	228.25 ± 27.82**	229.37 ± 28.67**	226.86 ± 27.06**	229.63 ± 30.27**
5th week	304.50 ± 37.19	234.25 ± 29.47**	239.56 ± 28.65**	232.28 ± 29.56**	238.51 ± 31.56**
6th week	328.60 ± 36.25	229.37 ± 35.07**	232.78 ± 38.53**	231.37 ± 33.61**	230.79 ± 37.25**

*Significantly different from SC, *P* < 0.05. **Significantly different from SC, *P* < 0.01.

**TABLE 3:** The CHr of rats after the experiments.

Group	CHr(pg)
SC	17.63 ± 0.16
EC	18.66 ± 0.35*
E + 1h	17.61 ± 0.17^##^
E + 2h	17.80 ± 0.31^##^
E + (1 + 1)h	17.52 ± 0.23^##^

*Significantly different from SC, *P* < 0.05. ^##^Significantly different from EC, *P* < 0.01.

**TABLE 4:** The Retic* and Retic% of rats after the experiments.

Group	Retic# (10^9^/L)	Retic% (%)
SC	183.98 ± 13.26	2.22 ± 0.17
EC	226.56 ± 23.93**	2.75 ± 0.19**
E + 1h	301.96 ± 97.31**^##^	4.17 ± 1.56**^##^
E + 2h	289.52 ± 121.30**^#^	3.97 ± 1.70**^#^
E + (1 + 1)h	321.52 ± 96.17**^##^	4.59 ± 1.53**^##^

**Significantly different from SC, *P* < 0.01. ^#^Significantly different from EC, *P* < 0.05. ^##^Significantly different from EC, *P* < 0.01.

As shown also in Figures 1A–C, the hematological parameters of all the IHE groups [E + 1h, E + 2h and E + (1 + 1)h] were comparable with those of the SC group (*P* > 0.05), indicating that hypoxic exposure elevates these parameters. Consistently, all the IHE groups displayed statistically significant difference on most of these parameters compared with those of the EC group (*P* < 0.05, Figures 1A–C) although their inter-group difference on these parameters were insignificant (*P* > 0.05). The RBC and Hct of the E + 1h group were two exceptions as they didn’t display statistically significant difference compared with those of the EC group (*P* > 0.05, Figures 1A–C). Furthermore, there was no significant difference between the EC group and the IHE groups in either food intake or body weight (Tables 1, 2). Finally, reticulocyte count and reticulocyte percentage of the rats in the IHE groups were significantly higher than those in the EC group (*P* < 0.05 or *P* < 0.01) (Table 4). This result revealed that the amount of reticulocytes was increased significantly after IHE, suggesting that IHE promotes erythropoiesis to increase the hemoglobin concentration. Together, these data indicate that 1-h IHE was sufficient to prevent the decrease of these parameters in the exercised rats and especially the appearance of low concentrations of hemoglobin.

As hypoxia can stimulate EPO synthesis that can then stimulate erythropoiesis, renal and serum EPO levels were examined. As revealed in Figures 1D,E, renal and serum EPO levels were not altered by exercise (*P* > 0.05, EC vs. SC). However, the renal and serum EPO levels of the IHE groups were significantly increased compared with those of the SC group (*P* < 0.05 or *P* < 0.01). Meanwhile, the renal EPO levels of the IHE groups were also significantly higher than those of the EC group (*P* < 0.05). On the other hand, the differences in serum EPO levels between the IHE groups and the EC group were statistically insignificant (*P* > 0.05). Together, these data suggested that IHE might stimulate the production of EPO to prevent intense exercise-induced low concentrations of hemoglobin.

## Discussion

Timely and sufficient recovery of physical function is key for athletes to adapt to intense exercise training (Kellmann, 2010). If the [Hb] drops too much after intense exercise training, it may indicate that the athlete’s physical function has not fully recovered and will soon display maladaptation (Zhao, 2003; Sheng, 2006). Currently, one strategy is to supplement athletes with oxygen to compensate their oxygen-carrying capacity that may decline and promote the recovery of their physical function (Sperlich et al., 2011). However, this strategy offers only a temporary solution, since it cannot fully restore exercise-induced low concentrations of hemoglobin in athletes. In contrast, our study tested a strategy that stimulates EPO synthesis and increases RBC and Hb production through moderate exposure of the rats to hypoxia, and that depends on an endogenous machinery in their bodies against the training-induced decreases in [Hb]. The long-term activation of this machinery may not only promote the recovery of physical function and the adaptation to intense exercise training, but also improve physical function and performance.

Hypoxic training was developed in the early 1990s in which the athletes are exposed to systemic and/or local hypoxia at rest (passive) or combined with exercise training (active) (Millet et al., 2010; Ramos-Campo et al., 2020). Currently, there are several strategies of hypoxic training and/or altitude exposure: “live high-train high” (LHTH), “live high-train low” (LHTL), intermittent hypoxic exposure during rest (IHE), and intermittent hypoxic exposure during continuous sessions (IHT) (Millet et al., 2010). Previous studies have revealed that LHTL and IHE (both use passive hypoxic exposures) are the two best strategies to promote Hb generation (Millet et al., 2010). The LHTL strategy was originally designed for athletes to improve their aerobic capacity; however, to promote the production of red blood cells it requires exposure to hypoxia for more than 12 h every day, which might not be feasible for many athletes (Millet et al., 2010). Conversely, methods of IHE (such as those adopted in this study) may be much more feasible. However, to the best of our knowledge, the IHE strategy has not been applied to promote adaptations to periods of the intense exercise training in athletes. This may be partly due to economic reasons such as the need for specialized equipment that can produce hypoxic environments. In the future, increasing the availability of relevant equipment may facilitate the application of the IHE strategy in promoting adaptations to intense exercise training in athletes.

Previous studies have subjected healthy athletes to simulated altitudes in excess of 4000m (Rodriguez et al., 2000; Fu et al., 2007). However, in the current study, we adopted a moderate simulated altitude (3000 m) based on our team’s previous research where we found that IHE (12.6% oxygen, simulated altitude of 4000 m) after intense exercise training led the myocardium of rats to partial decompensation, suggesting that irreversible injuries may occur in this protocol (Huang, 2003). However, there was no partial decompensation of myocardium when IHE was conducted at simulated altitude of 3000 m (Huang, 2003). Therefore, we chose 14.5% oxygen to in this study. Indeed, our data show that such a simulated altitude was sufficient to prevent low concentrations of hemoglobin in SD rats. However, laboratory animals, such as the rodents used herein, can be more sensitive to hypoxia than humans (Gonzalez and Kuwahira, 2018). Therefore, future experiments should test the efficacy of the simulated altitude and duration of hypoxic exposure from this study in humans with low concentrations of hemoglobin, but given the hypoxia-EPO-hemoglobin machinery is conserved in both humans and rats, similar findings may be expected (Haase, 2013; Viscor et al., 2018) (unpublished data for another manuscript in preparation). Furthermore, in this study, altitude was not simulated using hypobaric hypoxia whereby hypoxia is caused by reducing the barometric pressure, but normobaric hypoxia whereby hypoxia is caused by decreasing the fraction of inspired O_2_. As there may be differences in the physiological responses elicited by hypobaric hypoxia or normobaric hypoxia (Faiss et al., 2013; Saugy et al., 2016), whether hypobaric hypoxia can lead to a better adaptation to intense exercise than normobaric hypoxia deserves future study.

In this study, the differences in serum EPO levels between the IHE groups and the EC group were statistically insignificant (*P* > 0.05). Changes in serum EPO are dynamic, and it is difficult to predict the peak serum EPO during sample collection; therefore, one possibility that cannot be excluded is that the peak serum EPO did not appear at the time when the blood sampling was conducted. In addition, although the hematological parameters of most IHE groups increased significantly (*P* < 0.05 or *P* < 0.01) compared with those of the EC group, the E + 1h group did not display a significant elevation in RBC and Hct compared with the EC group (*P* > 0.05), indicating that 1 h of hypoxia exposure may not be sufficient to prevent the decrease of RBC and Hct in SD rats during intense exercise training. However, the RBC and Hct of the other two IHE groups, E + 2h and E + (1 + 1)h, were significantly higher than those in the EC group. This suggests that increasing the time (dose) of hypoxia exposure can more comprehensively improve the hematological parameters of rats with low concentrations of hemoglobin, although 1 h of hypoxia exposure was sufficient to significantly improve [Hb]. Beside, to increase the number of intermittent hypoxia stimulation, we designed the E + (1 + 1)h group in this study. Unfortunately, the hematological parameters of the E + (1 + 1)h group was not significantly improved compared with those of the E + 2h group. But this design deserves further exploration in future studies.

## Limitations

In this report, we showed that 1 h of normobaric hypoxia exposure (14.5% O_2_ per day from the fourth to sixth week) could prevent the rats from low concentrations of hemoglobin after exercise during six-week intense exercise training. However, lack of data regarding blood volume change in rats before and after IHE makes the possibility that increase in [Hb] in the IHE groups might simply reflect some hemoconcentration unable to be excluded completely, although the data of reticulocyte count suggests that IHE promotes erythropoiesis to increase the hemoglobin concentration. In addition, lack of data of the rats before the hypoxic intervention makes that the impact conferred by IHE on preventing from low concentrations of hemoglobin after intense exercise training unable to be evaluated from the intra-group level, although this report provided an evaluation from the inter-group level using the EC group as the control.

## Data Availability Statement

The raw data supporting the conclusions of this article will be made available by the authors, without undue reservation.

## Ethics Statement

The animal study was reviewed and approved by Guangzhou Sport University Animal Ethics Committee.

## Author Contributions

XW and WL conceived and designed the research. XW, HC, GX, and YM conducted the experiments. XW, HC, XY, and GM analyzed the data and wrote the manuscript. All authors read and approved the manuscript.

## Conflict of Interest

The authors declare that the research was conducted in the absence of any commercial or financial relationships that could be construed as a potential conflict of interest.
